# Editorial Note: The impact of city size on income inclusive growth: A human capital perspective and evidence from China

**DOI:** 10.1371/journal.pone.0349897

**Published:** 2026-05-28

**Authors:** The PLOS One Editors

The *PLOS One* Editors issue this Editorial Note because this article [1] was identified as one of a series of submissions for which we have concerns about aspects of the peer review process. Readers are advised to interpret the article [1] with caution.

The Editors have no evidence of any author involvement in the peer review concerns.

## References

[1] He S-L, Zhong Y, He W-W. The impact of city size on income inclusive growth: A human capital perspective and evidence from China. PLoS One. 2024;19(2):e0288294. doi: 10.1371/journal.pone.0288294 38346090 PMC10861076

